# “Hand-it-on”: an innovative simulation on the relation of non-technical skills to healthcare

**DOI:** 10.1186/s41077-016-0031-0

**Published:** 2016-12-05

**Authors:** Peter Dieckmann, Louise Graae Zeltner, Anne-Mette Helsø

**Affiliations:** Copenhagen Academy for Medical Education and Simulation (CAMES), Center for Human Resources, Capital Region of Denmark, Herlev Hospital, 25th floor, Herlev Ringvej 75, 2370 Herlev, Denmark

**Keywords:** Simulation, Gaming, Organizational change, Non-technical skills, Debriefing, Teams, Groups

## Abstract

Non-technical skills (NTS) are an integral part of the abilities healthcare professionals need to optimally care for patients. Integrating NTS into the already complex tasks of healthcare can be a challenge for clinicians. Integrating NTS into simulation-based training increases the demands for simulation instructors with regard to scenario design, conduct, and debriefing. We introduce a simulation game, *Hand-it-on*, that can trigger discussions on how NTS can influence work processes. *Hand-it-on* aims to help clinicians and simulation instructors alike to improve their understanding of NTS concepts and where they can apply them in their work. It complements existing approaches to teaching NTS by limiting the complexity of the game and by removing medical content, allowing learners to concentrate on NTS. *Hand-it-on* is relevant for groups and teams working across the range of different healthcare contexts. During *Hand-it-on,* participants stand in a circle and hand on everyday objects to each other according to simple rules, resulting in many events that can be debriefed in relation to safe patient care. We describe both the conduct of *Hand-it-on* and ideas on how to debrief participants. We provide variations that can be used in different contexts, focusing the exercise on different learning goals. We also offer the theoretical rationale for using an out-of-context simulation in combination with other forms of teaching. Although we did not evaluate *Hand-it-on* formally, oral feedback from participants and the replication of *Hand-it-on* by many simulation teams support its value.

## Introduction

Non-technical skills (NTS) are defined as “the cognitive and social skills that complement technical skills and medical knowledge in task performance” [1]. There are many connections between NTS and the care of patients [2, 3]. NTS can help to avoid errors or their negative consequences, for example when speaking up in the light of safety breaches [4]. Compromised NTS can have a negative effect on safety, for example in case of cognitive shortcuts [5, 6] or stereotyping [7] possibly resulting in fixation errors, barriers for inter-professional work, or the relation to patients. Studies show the positive impact of NTS on the care of patients [8, 9]. Several professional bodies describe NTS as an integral part of care [10–13]. We acknowledge the debate around the term NTS possibly implying a lesser value of non-technical than technical skills, using a negation to define a concept, and that NTS describe only a subset of the so-called “human factors” [14]. However, as it is widely established, we use the term for the purpose of this article.

Developing a deep understanding of the concepts underlying NTS is a challenge for healthcare professionals and simulation instructors alike [15]. Taxonomies of NTS are meant to facilitate their understanding and use. The “Anaesthetists’ Non-Technical Skills System” ANTS [16], a so called behavioral marker system, is such a taxonomy. ANTS describes observable behaviors related to NTS. It comprises four categories “Situation Awareness”, “Decision Making”, “Task Management”, and “Team-working”. Each category in turn contains three to five elements further describing the category. Each element is described by positive and negative behavioral examples. We use ANTS in this paper as an example for an NTS framework. It is widely used and stimulated the development of similar frameworks in different disciplines. However, *Hand-it-on* and its debriefing can be adapted to any framework describing non-technical skills [17–21].

### Training non-technical skills

There are also different teaching approaches to facilitate the learning of NTS and their practical application, simulations being a prominent example [8, 9, 22–27]. Game-like approaches are another example and are increasingly being used in healthcare for a variety of topics and within a growing field of disciplines [28–30]. We discuss the use of games in the next section in more detail.

We present a simulation-based game, “*Hand-it-on*”, that aims to complement existing approaches to support the learning of NTS. *Hand-it-on* uses simple, non-medical tasks combined in a way so that a complex pattern of interaction between the participants emerges. Participants stand in a circle and hand on objects to each other according to specific rules. The experience part of *Hand-it-on* provides the basis for the reflections during the debriefing, where most insights occur. During the debriefing the dynamics of *Hand-it-on* are analyzed and then related to actual healthcare. First, we provide a rationale for using out-of-context simulation games, here, *Hand-it-on,* in healthcare education. We then describe its typical conduct, along with possible variations and their purpose. We provide ideas for the debriefing. Lastly, we describe some typical take-home messages. We aim to stimulate the replication (and evaluation) of *Hand-it-on* by others.

### Rationale for using *Hand-it-on*

Conceptually speaking combines *Hand-it-on* elements of simulations and games. It enables generation of learning opportunities in a simulated situation (participants, simulate working in an “organization” and engage in simulated tasks), without endangering any patients. Its game-like character draws on several learning principles and makes use of certain learning mechanisms [31]. We first discuss the principles: *Hand-it-on* generates *intrinsic motivation* as most people perceive it as fun to play it. While the tasks participants perform are artificial, participants can (in our experience) easily see them as a representation of tasks they do in clinical care, especially during the discussion in the debriefing. *Hand-it-on* provides ample opportunity for learners to try out different behaviors and supports such an approach by its playfulness. Finally it is a highly experiential learning situation, which is combined with reflection in the following debriefing. Those principles are implemented in practice via a range of mechanisms [31]. There are rules for the actions of participants and (vaguely) defined goals. *Hand-it-on* is constructed as a fictional setting (an organization is simulated) that provides some background information for the tasks to be done. It is possible to implement increasing levels of difficulties (see the discussion of possible variations below). There is a high degree of participant interaction as well as some elements of surprise and uncertainty. There is feedback while the game is running—from the other players, from the processes themselves, and from the game leader. There is also feedback and reflection during the debriefing. Finally, *Hand-it-on* is a highly social group learning activity. Research shows that those principles and mechanisms are related to high engagement of learners [31], while the evidence that this results in changed behaviors or even improved outcomes are still sparse [30, 31].

By removing medical content and basing *Hand-it-on* on the straightforward task of handing on everyday objects from one person to another, participants can concentrate on the essence of what is occurring during the organization of the processes. Many other simulation modalities, such as manikin-based simulations, challenge participants on three different dimensions of complexity [32]: the actions taken during diagnosis and treatment of the depicted patient; the issues from using the simulator as a complex technical device; and the issues of NTS and how they relate to patient care. The combination of all three aspects can burden participants’ learning [32, 33]. *Hand-it-on* removes the medical challenges and uses simple tasks, reducing complexity from the task itself and from using the simulator as device and setting the focus on the third, namely, NTS in their relation to healthcare. These connections can be discussed in the debriefing.

Working with such out-of-context exercises can remove threats to participants’ self-image as a clinician. For most, there is little at stake as a healthcare professional when handing on objects to another person. Typically, none of the participants is an expert at the tasks involved, thus the existing order in hierarchy and/or expertise of the participants is less relevant during the game’s conduct.

*Hand-it-on* caters to different learning styles [34]. The highly active and physical nature of *Hand-it-on* renders the game particularly suitable for learners who prefer active engagement during learning. The physical aspects also support the learning on a more bodily plane, which, for example, was shown to support the acquisition of concepts in different educational contexts [35]. An in-depth discussion of the activity during the debriefing caters to participants with a more theoretical-learning orientation [34].

Apart from the cost regarding the time and remuneration of those involved and of that of securing a venue, *Hand-it-on* is virtually cost free. Most of the equipment is inexpensive and can be re-used.

### Challenges related to the transfer of learning

However, due to its abstract nature, *Hand-it-on* requires some translation between the dynamics that unfold during its conduct and the dynamics in actual workplaces. It is not the intention that participants learn how to better hand on objects to each other. The intention is that participants acquire knowledge and skills that they can use in their clinical practice. We aim to create reflection on clinical practice and participants’ attitude towards NTS and safety in organizations. *Hand-it-on* requires the so-called “far transfer” [36, 37]. Participants and instructors need to discuss connections between what happens during the exercise and what happens in clinical work. An example is dealing with unclear instructions. Some of the instructions for *Hand-it-on* are purposely vague. During the debriefing, the implications of those vague instructions for the conduct of the game are discussed. The group can then compare those implications with unclear instructions in actual healthcare. By experiencing how to handle unclear instructions in *Hand-it-on* and by discussing this aspect in the debriefing, participants can develop ideas on how to improve this aspect in clinical care. By requiring participants to act in the unfamiliar context of *Hand-it-on*, NTS can become evident in a new light, possibly triggering deeper reflections on their meaning [38]. One known strategy to support the learning of complex concepts is to discuss examples of such concepts in different contexts [35]. Table 1 shows human factors related references that supplement the ANTS categories and provide some guidance on what to note during the conduct of *Hand-it-on in relation to the categories*.

**Table 1:** Non-technical skills (NTS) categories and examples of literature relevant for the conduct and debriefing of *Hand-it-on*

ANTS categories [16]	Underlying and related concepts and further references	Examples of relations to *Hand-it-on* and participants’ behavior
Situation awareness	• Situation awareness is composed of the three elements: perception, comprehension and projection into the future [46]. • The concept of situation awareness as a single variable was challenged and its division into sub-dimensions requested [47].	Perception: • Who recognizes the workload of other team members? • Do all participants hear the instructions given or ideas that come from team members? Comprehension: • How do the different participants involved interpret the instructions given? Projection: • Does the group anticipate further challenges from more processes? • Do they discuss obstacles to the implementation of improvement ideas during a debriefing round?
Decision making	• Decision making can be analyzed from a more analytical or from a more intuitive angle [48]. • Processing numerical information to reach sound decisions depends to a large extent on how the information is presented and rules of thumb often help in this process [6].	• What kinds of decisions were taken during the simulation (e.g., to assign a leader)? • What kinds of criteria were considered when making these decisions? • To what extent was the decision process analytical vs. intuitive? • Was there any numerical information used? • Were all participants aware of the decisions taken?
Team working	• Co-ordination behavior in a team can be described along two dimensions in care situations: “explicit vs. implicit” and “actions vs. information” [49].	• How explicit is the information shared in the group and/or between the two groups in the version for two groups? • Is the co-ordination more action-oriented or more information-oriented? • How does the co-ordination pattern change over time? • What verbal cues are used in the co-ordination process? What non-verbal cues were used? • What is the relationship between plans and their implementation?
Task management	• Task management needs to be adjusted to the different context and the persons involved adapt their actions to the changing dynamic of the situation [50].	• What kinds of adjustment are made (e.g., establishing a “task force” for the unexpected event)? • What triggers such adjustments (e.g., task overload, time, discussion)? • What are obstacles to implement such changes (e.g., slowing down of the core process)?

## Considerations before using *Hand-it-on*

The target group for *Hand-it-on* is diverse. Healthcare professionals from all disciplines, specialties, and experience levels can participate. *Hand-it-on* is suitable for people working in the various group and team constellations in healthcare, whether ad hoc groups, fixed teams or other constellations. *Hand-it-on* can be used in courses for healthcare providers and also in faculty development programs. The conduct and debriefing of *Hand-it-on* can be adapted to focus on the constellation most relevant for any particular setting.

The optimal number of participants for *Hand-it-on* is 9–13. With fewer participants, the tasks become easier, participants are then less challenged and may therefore not fully engage; more participants can be included in observer roles or in the variation for larger groups, described below.

By its nature, *Hand-it-on* can be used with different focus points. Table 2 describes possible learning goals; for each conduct of the game, one or two are typically chosen. We assume that instructors using *Hand-it-on* adjust it to their context and that they value experiential learning. Instructors should have at least a basic knowledge of the NTS concepts. This makes it easier to recognize relevant events during the game and to use those observations during debriefings [39]. Tables 3 and 4 provide ideas for observing the conduct of *Hand-it-on* and its debriefing.

**Table 2:** Possible learning goals for *Hand-it-on*. Typically, one or two are selected for any given conduct

After taking part in *Hand-it-on* participants should be able to:
• Describe their actions and observations during *Hand-it-on* using a previously introduced NTS framework, for example ANTS [16]. This could be investigated ad hoc by using the instructor’s impression or systematically via transcripts of the debriefing.
• Describe possibilities in their clinical practice, where they might use NTS elements to improve patient safety and quality of care. This could be analyzed via interviews.
• Replicate *Hand-it-on* in settings in which they teach. This could be analyzed by observing how participants run *Hand-it-on*.

**Table 3:** Observable events during *Hand-it-on* and examples of relations to clinical practice

Observable events during the simulation	Examples of relations between the observations and events in clinical settings
Sequence errors, where an object is passed to the wrong person.	• Omitting a step in an algorithm, such as the ABCD approach. • Lacking a piece of information during a handover situation.
Dropping objects.	• A technical error in the procedure, for example, perforating a vessel while placing an intravenous access.
One participant holds more than one object at a time.	• A leader who tries to coordinate the tasks in the team while ventilating the patient manually. • A nurse who receives more than one request at the same time.
Little or no verbal communication, for example, not using names.	• Not addressing a member in a trauma team directly but asking *someone,* so that the message does not reach the intended person.
Participants throw objects without caring whether another person can catch the object.	• Asking an orderly to fetch something from a different room and ignoring the objection from that person that it is not part of the agreed job description. This creates a dilemma arising from the conflicting conditions of the official job regulations and the current, pressing, social environment.
Different participants assign different relative importance to the objects in the simulation (and are unaware of doing so).	• Different priorities in the treatment based on the highly specialized views of those involved, representing different professions and disciplines.
Different participants have different understandings of the speed vs. accuracy trade-off across the objects [51]: Some try to hand on things quickly; others try not to make any errors.	• Handover situations between colleagues where the attempt to use the Situation, Background, Assessment, Recommendation (SBAR) [52] structure is interrupted by the request to concentrate on the key issues only.
Questioning the task and the priorities with—often unclear—questions to “senior management” and continuing without getting clear answers.	• Change processes in a department where the goals of the change and the manner of its implementation are not communicated clearly.
Jokes and “play” in the beginning as the simulation is still slow.	• Trying different approaches in treating the patient while the workload is low. • Being tricked into a lower level of alertness while the patient’s problem seems easy, possibly resulting in an “everything OK fixation error” [20].
Systematic variation of passing on the objects, for example, trying different hand positions to make it easier to receive the objects.	• Systematically varying the way that a new intravenous needle is manipulated to get a feeling of its characteristics.
Helping each other by correcting errors, for example, by pointing out that another person should receive an object.	• Mentioning to a leader that the medication he/she is about to request has already been administered.
Deliberately making it difficult for each other to receive the object.	• Not mentioning that a piece of equipment requested has arrived, because the colleague asked in a harsh tone for it.
Throwing objects out of the circle.	• Ignoring the request by a younger colleague to get some feedback about his/her performance in a certain procedure.
Assigning certain people to handle the “unexpected events”	• Establishing Medical Emergency Teams in an organization.
Establishing some kind of rhythm that helps in the pacing of the exercise, for example, memorizing which object is received from which person and to whom it needs to be passed.	• Establishing a habitual information flow pattern in departments, whether by written or oral agreement.

**Table 4:** Possible focus points for the debriefing of *Hand-it-on*

• Ask participants to describe what events occurred during *Hand-it-on* (e.g., things being dropped, wrong sequence, little talking, confusion). Collect such descriptions until “complete” and possibly take notes on a flipchart. Initiate a reflection on the differences in participants’ perceptions and memories. Discuss relations of the issues observed to clinical practice (e.g., “Where do you experience ‘wrong sequences’ in your clinical practice? See Table 1 for inspiration). Investigate the basis for the relevant events (“What triggered you to start using names before passing objects?”).
• Ask participants to relate the issues of *Hand-it-on* to non-technical skills and their relation to healthcare, for example, by asking them to sort what happened into the ANTS categories [16] or the principles of crisis resource management [20]. Correcting a colleague who passes to the wrong person can be seen as an example of “Use Good Teamwork”. Other participants might see the same act as an example of “Leadership and Followership”. The differences in such interpretations can stimulate discussions that aid participants’ learning around the concepts involved [35]. Differences in interpreting the efficiency/accuracy trade-off [51] can be used to discuss “Set Priorities Dynamically”.
• Ask the participants what the core processes, routine tasks and unexpected events in their organization are. Discuss any different views relating to these questions or uncertainties in finding an answer. This would be especially interesting when working with actual work teams.
• Ask the participants to identify the strengths of what happened in the organization and analyze what helped to create them [5]. For example, what facilitated correcting a colleague who made a wrong decision? Further, discuss the consequences of weak points. For example, why was there little communication in the beginning and what were the effects on the processes.
• Ask the participants to reflect on their technical skills and how those are acquired and refined over time. Participants often experiment, more or less consciously, with different movements while handing on objects. For example, how did you learn to find a good way of passing on a small coin? What did you learn from observing others? What could you acquire only by performing the task yourself? Are there parallels of learning in handing on an object to, for example, learning how to intubate?
• Ask the participants to discuss the effects that improvement initiatives had on the different processes. Typically, there is a marked drop in speed when the group tries to `optimize any aspect of the processes (e.g., combining the unexpected events with the routine tasks or starting to use names). Discuss how any changes in a group have an impact on what happens to routine tasks.
• Ask the participants to reflect on violations of procedures [53], for example, whether participants make “fun” of the tasks or try to trick their team members by making it difficult to receive the objects (for example, by purposely not looking at them when handing them an object). Discuss differences between the levels of stimulation people seek in their jobs and how willingly they accept rules.
• Ask the participants to discuss any kind of role distribution during *Hand-it-on* and how these roles were assigned and how clearly they were accepted. Who helped others? Who concentrated mostly on their own tasks? What are the advantages and disadvantages of such different focus points? Are such roles known in the participants’ organizations? Do the different crews involved in treating the patient actually become treatment teams? [54] Are those persons aware of the effects they have on the organization? Do they want to have these effects? How do their colleagues react to them?
• Ask participants to reflect on the challenges to really understand how organizations function [55]. The simple simulation presented here can illustrate this point by generating complex patterns of interactions from a combination of very simple tasks. Ask participants to draw parallels to their actual work settings and the much higher complexity there. This insight might enable participants to better appreciate the complexity of their work systems. It might create new ways of thinking about how to interact with those systems and how to relate to colleagues who might also struggle with fulfilling their tasks with the given complexity of the system.
• Ask participants to discuss the many idiosyncratic ways of interpreting the instructions and aligning them according to different personal priorities. Who was trying to work fast, for example? Who tried to avoid any errors? Were participants aware of different interpretations by their colleagues?
• In the debriefing of the variation for two large groups, explore the respective perceptions of each group. What did they do? How were these actions interpreted by the other group? How is the other group seen? To what extent was each group aware of what was happening in the other group? The events provide rich material for discussion about the relation to “clinical practice” and different professions, specialties, departments, hospitals: How do “the anesthesiologists” see “the surgeons”, how do the “clinicians” see the “administrators”? How relevant are those relations for the patient’s treatment and the interaction with his or her relatives? Be prepared for what could be a surprisingly strong group dynamic between the groups.

### Learning atmosphere

It is essential that instructors thoroughly brief participants to establish a learning atmosphere [40, 41]. This could include providing the rationale for the exercise and establishing ground rules. Further relevant theoretical background could be provided, such as a discussion of the ANTS [16] categories and elements.

Any learning setting requires a certain amount of “psychological safety” [42, 43]. In our experience, this game feels very safe for participants, despite some of the instructions being vague. While such vagueness could potentially threaten psychological safety, *Hand-it-on* seems to be sufficiently “game-like” for participants to perceive the vagueness as a trigger for discussion and reflection rather than as a threat. Humor can help in setting the scene for *Hand-it-on*.

Nevertheless, some participants might not perceive the relevance of such a “game”. We have seen that participants accept the value of *Hand-it-on* if a mutual rapport is established between the instructors and them and if the instructors themselves are convinced of the value of *Hand-it-on*. Providing this is the case, instructors will be able to transport this conviction to the participants. The arguments provided in the introduction of this paper (avoiding cognitive overload, focussing on NTS, increasing psychological safety by removing healthcare content, addressing of different learning styles, low cost) can also be used to support the use of out-of-context exercises.

### Logistics of implementing *Hand-it-on*

The typical duration of *Hand-it-on* is about 50–60 min, including: (1) briefing participants and running *Hand-it-on*; (2) doing a debriefing; (3) running *Hand-it-on* again; and (4) leading a second debriefing. Depending on the depth of discussion, the duration can be extended to 90 min or longer.

To conduct *Hand-it-on*, apart from participants, three objects are needed. The objects should not break if they are dropped and should not cause injury. Examples of objects are a ruler, a coin, and an inflated balloon. There should be enough space for participants to stand in a circle and extend their arms without touching each other. A flipchart or whiteboard for notes during debriefing can be helpful.

## The conduct of *Hand-it-on*: the guide for instructors

**Fig. 1:**
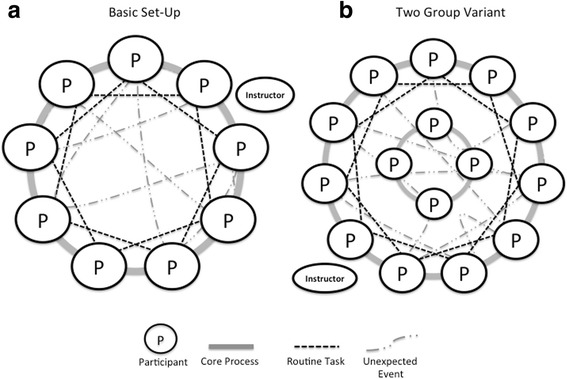
Basic set-up of *Hand-it-on* (**a**) and the “two-department variation” (**b**). The larger “group” should have an uneven number of participants to involve all participants in the routine task. Each *circle* represents a participant in *Hand-it-on*, the ellipse the instructor. The *lines* represent the movements of the different objects: Core process: handing an object to the immediate neighbor. Routine task: handing another object to every second person. Unexpected event: no defined pattern of handing on

The participants stand in a circle and simulate “an organization” with a set of different tasks. According to specific rules, the tasks are simulated by handing objects to each other (Fig. 1). While passing objects, many events typically occur that can be debriefed (e.g., incorrect passing order, helping a colleague decide what to do, objects being dropped, optimizations of the work). The concrete goals selected for any conduct of *Hand-it-on* will determine what to focus on during the observation (Tables 1 and 2). More than one round can be run, so participants can experience differences in their performance after debriefing.

Conducting *Hand-it-on* is based on the following steps (see Fig. 1a):

1. Prepare participants for a basic understanding of NTS (e.g., pre-course reading assignment or a discussion of NTS).
2. Set the scene by establishing concrete learning goals for the session. Describe how such an out-of-context simulation can be useful.
3. Throughout the exercise observe what happens and possibly take notes.
4. Prepare the objects and keep them out of sight until they are to be used.
5. Ask participants to stand in a circle so there is sufficient room for them to stretch out their arms to their sides without touching each other.
6. Stand outside the circle next to a participant. Do not take part in the handling of the objects after passing them into the “organization”. Explain: “We are simulating an organization. I am senior management in your organization. Every organization has a core process. The core process in your organization is to pass this ruler from one person to another.”
7. Hand the ruler to the person next to you and indicate with your hand the direction in which the ruler should be passed by pointing at the next three persons to receive it. Say: “The core process is running all the time, so please continue with this process throughout the exercise.” Correct the group if needed.
8. Let the group pass around the ruler for about two rounds.
9. Explain while the group continues: “Besides the core process, there are also *routine tasks* in your organization. The routine task in your organization is to hand on this coin to every second person. It also runs all the time.” Hand the coin to the person next to you and indicate who should receive it. Correct if needed.
10. Let *Hand-it-on* run for about four rounds of the core process (ruler), while the routine task also runs in parallel.
11. Fetch the balloon and stand with the same person again. Say: “You know that there are sometimes unexpected events. Usually, you try to get rid of them as soon as possible”. While you say this, you hit the balloon so it flies to a participant as far away as possible. It is important to demonstrate the movement of the balloon because participants will typically mirror the action.
12. Allow the exercise to run for approximately three rounds of the core process, with the routine task and the unexpected event in parallel and then stop for the debriefing.
13. If time allows, start another round of *Hand-it-on* after debriefing. Ask participants to develop ideas on how to “keep what was good and to improve what could be improved” in relation to the learning goal discussed. Observe changes compared with the first round. Further, observe how many of the intended changes discussed during debriefing were implemented in the actual re-conduct of the game. Consider varying the “clarity” of priorities between rounds: Senior management could request that the objects are passed on more quickly or that no errors occur. Debrief again after such an additional round.
14. If an object, typically the balloon, falls outside the circle, retrieve it and pass it into the circle again. If participants combine objects (e.g., handing the balloon around together with the ruler), decide whether it is acceptable or correct as necessary—the learning goals for the session will help in making this decision. Note the size of the circle because it tends to get smaller, making the tasks easier. Adjust the circle size, if necessary.

For larger groups (approx. 20 to 30 participants), split the group in two. One group is organized as previously described, the other—consisting of four or five participants—stands in the middle of the larger circle. These participants stand close to each other (Fig. 1b); they are assigned only one task, but one that requires concentration, such as handing on a full glass of water. As this group will concentrate on this task only, it is less critical to have an object that might break, if it falls down. The purpose of this variation is to discuss the relationship between different parts of an organization. Here the two “departments”, the inner and the outer circle, are engaged in similar tasks that are, however, to be performed under different conditions. The three tasks in the outer circle are performed more dynamically and quickly, performed in a larger group where not all members can clearly see each other because the inner-circle members are obstructing their view. The inner circle has one task only, but one that requires concentration. It is much slower and can soon become boring. As there are only few people in this inner circle, all are directly involved and all are “seen” by others. To some extent, the inner group stands in the way of the outer group, especially when dealing with the balloon. The differences in group composition and tasks make interesting points for the debriefing, for example in relation to stereotyping of members of other departments, patient groups, etc. [7]. An additional advantage of this variation is the possibility to engage larger groups.

Other variations of the conduct of *Hand-it-on* are described in Table 5. Each variation emphasizes certain learning opportunities.

**Table 5:** Possible variations of *Hand-it-on* to emphasize certain learning goals

Variation	Purpose of the variation and ideas for the debriefing discussions
Include observers.	• Include an outside view on the processes during the conduct into the debriefing. What does such an outside view contribute to the analysis of the conduct of *Hand-it-on*? • Prepare participants for the observer role during scenarios and debriefings. What is helpful feedback to colleagues after a simulation scenario?
Vary the objects.	• Use objects where there is something at stake for those who handle them. When handling a full glass of water there is a different “risk” for the person handling the water than when handing on a pen. Where are own risks during the care for patients? How do they impact the actions of those involved? • Use objects that are difficult to manipulate (e.g., a thin thread). They can be used to focus on technical skills and psychomotoric challenges in combination with keeping an overview of parallel tasks. How does the requirement to concentrate on handling this object impact the overview of the situation?
Vary the direction in which the objects should be passed and/or include more tasks.	• This variation can challenge improvement plans between two rounds of *Hand-it-on*. How are the intentions to implement changes (work as imagined) impacted by the practicalities of the work system (work as done)? • Discuss the impact of work procedures and personal preferences on their implementation. How do changes in procedures impact the patient care?
Separate one or more participants while giving the details of the instruction. Let the first few rounds run without them and include those participants only then.	• Discuss the integration of new colleagues into a department. How are they introduced to the tasks they are supposed to do? How are they welcomed on a social and emotional level? What influences their integration (think about time pressure, work-load, or structured introduction programs)? Are there differences in the introduction between the two departments in the variation for large groups (See Fig. 1b)? • Discuss the importance of knowing about the history of discussions in a department. How do people understand a situation if they know only parts of its development and did not have any prior practice of the task? • Discuss working with people who might not have been part of forming the current routines, norms, values and beliefs. How does the shared understanding of those impact the task and social level? How does it feel to accept the features of the work system if you were not a part in forming them?
Include “hidden” instructions for briefed role players. You could ask them to frequently “drop” objects or to not concentrate on the process, causing delays. Remember to reveal the hidden instructions during the debriefing to protect your role player. They would not like to come across as “obstructive” or “unable” in reality.	• Discuss how the group reacted to such a “low” performer. How can a work team or an organization deal with a worker who does not perform to standards? How would a team identify such a person in the first place? What would impact the reaction to such a worker? Is there a difference in how you treat people who you like vs. those you do not get along with? How does it feel to observe this person? How does it feel to be this person? What do patients think about the low performer?
Include “stressors” such as loud music, noise, or the threat of moving the organization to a different country.	• Discuss the impact of such disturbances and ask participants to draw connections to real life. How do different operation room settings with their lighting, temperature or noise conditions impact performance? • Explore the differences in the experience and in handling of such stressors between the individuals involved. Who is disturbed by such external factors, who can deal with them easily? What are the coping strategies?

Note that the instructions for *Hand-it-on* are intentionally vague to allow participants to interpret them as they think fitting: It is not clear what role “senior management” has. It is also unclear how relatively important the different tasks are to each other and which should be prioritized. There is no statement concerning any speed/accuracy trade-off: should participants work as error-free as possible or should they try to hand on the objects as quickly as possible? There are no prompts regarding role distribution. This vagueness generates much of the dynamic of *Hand-it-on* and also reflects the vagueness of many of the real-world tasks people are assigned. Combined with the game character of *Hand-it-on*, this vagueness triggers extensive reflection, which can be analyzed in the debriefing.

*Hand-it-on* will spawn many issues for discussion during debriefing and these can be used to create learning opportunities for the participants regarding the set learning goals. Table 4 shows typical examples of observable events and relates them to possible applications in healthcare. Participants can usually find numerous examples from their own practice to relate to what happened during *Hand-it-on.* Table 4 provides debriefing ideas.

## Debriefing

Any simulation scenario and debriefing are embedded in the context of a simulation setting [40], for example, a training course. The course comprises different phases, such as the introduction, theory modules, scenarios, debriefings, and a course ending. Those phases influence each other and are all important in preparing for the best possible debriefing [40]. Here, we assume that relevant concepts had been discussed before conducting *Hand-it-on*, for example, a discussion of NTS. Displaying the ANTS categories and elements in poster format could also support the debriefing. We further assume that the instructor collected interesting observations during the conduct of *Hand-it-on.*

The debriefing can be run in different ways, depending on the goals chosen for the session and depth of discussion. Typically, it takes a minimum of 15 min but can easily be extended to 45 min or longer. The flexibility of the timing is another advantage of using *Hand-it-on.* In our practice, the group usually remains standing for the debriefing. This seems to support the active nature of *Hand-it-on* but might not be received well by those who find standing for prolonged periods difficult. In this case, the setting can be adjusted and those who wish to sit down can. Our approach is to stimulate and steer the discussion leaving most speaking time to participants [39, 44]. Depending on the group, we would emphasize either the game-like character of the exercise or the seriousness of the learning opportunities it creates. The first option would help to relax the group, whereas the second would help people who question the seriousness of the possible learning. We described further ideas for the debriefing in Tables 4 and 5.

We typically build our debriefing on four phases: setting the scene for the debriefing with a description of the debriefing model and the time frame; asking participants to describe what happened during the game in the description phase; analyzing what happened during the game in more detail according to set learning goals or other focus points (see Tables 4 and 5 for ideas) in the analysis phase; and asking participants to formulate take home messages in the application phase. In the description phase the emphasis lies on the description: what phenomena could be observed (see Tables 1 and 3)? In the analysis phase, we focus on how those phenomena came to be within the context of the game and ask participants to relate the phenomena and their explanations to the actual work settings and influences there. For example, it might be observed that in the variation for large groups, the members of the two departments make “funny” comments about the members of the other departments. The observations could be described in the debriefing, prompting a discussion of what triggered them. Finally, it could be discussed with the groups what kind of “funny” comments between departments (professions, disciplines, etc.) are made in real work settings and how the comments impact the care of patients. The discussion could be related to stereotyping [7] or other relevant concepts. Tables 4 and 5 provide numerous ideas on how to structure the discussion in the analysis phase. In the application phase, we try to help participants formulate take home messages as concrete as possible.

## Discussion

### Potential risks and limitations

We did not formally evaluate *Hand-it-on.* Oral feedback from sessions where we conducted it was positive and several groups have adopted the game into their teaching practice. We discuss possible risks and limitations in the following section.

Conceptually speaking, *Hand-it-on* can pose a risk if instructors wrongly interpret participants’ actions and consequently provide incorrect or incomplete feedback during debriefing, or if instructors do not challenge wrong interpretations by the participants. This risk could be lessened if faculty members familiarize themselves with the NTS concepts and facilitation techniques.

*Hand-it-on* might favor people with active learning styles who are “game-minded”. Not all participants will be able to easily see the connections between handing on objects to each other and working in healthcare settings. Here, the debriefing is paramount. Instructors can facilitate the discussion of such connections.

It would be interesting to investigate *Hand-it-on* in terms of cognitive load theory, distinguishing different types of cognitive load. *Intrinsic load* describes the complexity of the topic to be learned; *extraneous load* describes the complexity of the material used to support the learning; and *germane load* relates to cognitive activities connected to the processing of information [33]. While a full conceptualization of *Hand-it-on* based on cognitive load theory is beyond the scope of this paper, the game seems to trade-off low intrinsic (simplex tasks) and extraneous load (simple rules) with a high germane load that stems from the need to “far transfer” [36] the learning to the clinical setting.

### Lessons learned

To date, we have run *Hand-it-on* in approximately 25 occasions, reaching about 500 participants from a broad range of backgrounds. We have met them in different settings, including conferences and faculty development programs. Several faculty development groups across the world have adopted *Hand-it-on*. We see this as a “proof-of-concept”. We now describe some of the key learning messages from our practice with *Hand-it-on* to further support its value as a complement to other teaching methods. They are based on our impressions during the conduct of *Hand-it-on* and how we remember discussions during debriefings. They are not based on scientific investigations.

*Hand-it-on* can pave the way for intense discussions in the debriefing. One discussion topic concerns the difference between “work as imagined” and “work as done” [5]. What is assumed about how a group works in an organization (work as imagined) is not necessarily what actually happens (work as done) and *Hand-it-on* helps participants understand this difference. When re-started after debriefing, *Hand-it-on* can illustrate that not all planned intentions to optimize a process (work as imagined) will work in practice (work as done). This is seen, for example, when participants discover that they assigned different priorities to the intended changes and that only some participants implemented the changes, despite all having agreed to them.

Another focus point for the debriefing of *Hand-it-on* can be how participants produce good practice—an important aspect of modern safety theories, such as Hollnagel’s concept of Safety II [5]. The debriefing can focus on how the participants made sure that no sequence errors occurred. How can these ideas be applied to more complex clinical settings? Concentrating on such process aspects is facilitated in *Hand-it-on* as the complexity of the content is low.

The discussions after *Hand-it-on* can illustrate the connection between the NTS and the care of patients: established “technical” procedures (passing sequence or the movements that are used to hand on an object) might not be known to all involved (situation awareness), might not be accepted by all (leadership and followership), might be found by chance after non-systematic considerations (decision making), or might be difficult to implement while keeping the core process running (task management).

*Hand-it-on* can also be used to reflect on the influence of emotions on people’s behavior. Not all that happens can be explained by attempts to hand on the objects/to optimize the care of patients and their relatives. Some actions are done to avoid boredom, to “spice things up”, or for other egocentric motives. Nance et al. describe how this can unfold in clinical practice in a very illustrative way [45].

## Conclusion

We describe an innovative simulation-based game, *Hand-it-on*, that uses simple tasks to illustrate the connection between NTS and care of patients. By removing medical content from *Hand-it-on*, we aim to focus on NTS and their role in healthcare. The features of the game, seen from a theoretical viewpoint, are intended to support the learning on the level of principles that can be applied in different settings. The events that occur during *Hand-it-on* can be used to create learning opportunities around the interplay of technical skills and NTS. Anecdotal evidence from running *Hand-it-on* for approximately 500 participants over the recent years supports the perception of positive effects of the exercise. Whether those effects translate into improved care remains to be investigated.

## Abbreviations

CAMES: Copenhagen academy for medical education and simulation
NTS: Non-technical skills
